# Mapping the Racial Inequality in Place: Using Youth Perceptions to Identify Unequal Exposure to Neighborhood Environmental Hazards

**DOI:** 10.3390/ijerph13090844

**Published:** 2016-08-25

**Authors:** Samantha Teixeira, Anita Zuberi

**Affiliations:** 1 Boston College School of Social Work, 219 McGuinn Hall, Chestnut Hill, MA 02467, USA; 2 Department of Sociology, Duquese University, 519 College Hall, Pittsburgh, PA 15219, USA; zuberia@duq.edu

**Keywords:** environmental justice, neighborhood, poverty, youth, inequality, race

## Abstract

Black youth are more likely than white youth to grow up in poor, segregated neighborhoods. This racial inequality in the neighborhood environments of black youth increases their contact with hazardous neighborhood environmental features including violence and toxic exposures that contribute to racial inequality in youth health and well-being. While the concept of neighborhood effects has been studied at length by social scientists, this work has not been as frequently situated within an environmental justice (EJ) paradigm. The present study used youth perceptions gained from in-depth interviews with youth from one Pittsburgh, Pennsylvania neighborhood to identify neighborhood environmental health hazards. We then mapped these youth-identified features to examine how they are spatially and racially distributed across the city. Our results suggest that the intersection of race and poverty, neighborhood disorder, housing abandonment, and crime were salient issues for youth. The maps show support for the youths’ assertions that the environments of black and white individuals across the city of Pittsburgh differ in noteworthy ways. This multi-lens, mixed-method analysis was designed to challenge some of the assumptions we make about addressing environmental inequality using youths’ own opinions on the issue to drive our inquiry.

## 1. Introduction

Black youth in the United States are much more likely than white youth to grow up in poor neighborhoods and to face the double disadvantage of being from a poor family and living in a poor neighborhood [1,2,3]. In 2000, 45% of poor black youth were living in distressed neighborhoods compared to only 5% of poor white youth [2]. This stark racial inequality in the neighborhood environments of youth not only limits their access to high-quality schools and services, but also increases their contact with hazardous neighborhood environmental features including violence, physical and social disorder, and toxic exposures which contribute to racial inequality in youth health and well-being [4,5,6,7,8,9]. While a great deal of research has examined how the neighborhood environment affects youth, less has focused on how individual young people perceive, engage with, and “make meaning” of neighborhood-level environmental disparities [10]. This study aims to address this gap using a mixed-methods approach and an environmental justice (EJ) framework to explore what aspects of the neighborhood built and social environment matter for youth well-being.

Neighborhood environmental disparities have been characterized as “slow violence”; a phenomenon that exerts its injurious impact more gradually than traditional acts of violence but has similar enduring effects on its victims [11,12]. Research suggests that among blacks, the slow violence of poverty and deleterious social/institutional contexts is passed through generations [3]. In addition to broader structural issues, subtler environmental factors that are difficult to capture using traditional surveys may be contributing to neighborhood inequality [3,13,14]. These “subtle factors” manifest in vast differences in the experiences of neighborhood poverty among black and white Americans. Sampson and Wilson [15] summed up these stark contextual and racial differences by suggesting that, “The worst urban context in which whites reside is considerably better than the average context of black communities”.

While the concept of neighborhood effects has been studied at length by social scientists, this work has not as frequently been situated within an EJ paradigm. The existing work on environmental injustices and children has focused largely on toxic exposure (e.g., lead) and its disparate effects on children’s developing bodies [16,17,18,19,20]; toxic settings that children inhabit within their neighborhoods (e.g., schools) [8,21,22]; and on organizing with youth around traditional environmental issues such as pollution and its relationship to health outcomes like asthma [23,24,25]. Situating neighborhood environmental disparities as an EJ issue expands beyond traditional environmentalism, which focuses primarily on the preservation of rural, remote natural environments, to recognize that many urban issues are reflective of environmental injustices [26,27]. An EJ framework allows us to examine the neighborhood environmental disparities faced by black youth as one part of an intersection of oppressions that include poverty, spatial segregation, and exposure to environmental hazards; all of which have implications for health and well-being.

In order to better understand these subtler neighborhood features related to inequality, the present study builds on prior work by using youth perceptions to delve into more granular and potentially more malleable-aspects of the built and social neighborhood environment, identified by youth from a disadvantaged community in Pittsburgh.

We use qualitative data from a sample of black youth living in a disadvantaged neighborhood to identify aspects of the neighborhood’s built and social environment they deem important to their well-being, and then use quantitative data to map these indicators across all neighborhoods in the city of Pittsburgh with particular attention to how they are spatially concentrated by race. The focus of the manuscript is on environmental features defined by the youth in our qualitative sample. The youth focused on the immediate physical environment, rather than factors such as pollution and exposure to toxins; a trend noted in other studies, perhaps attributable to the perception that it is more distal than visible aspects of neighborhoods [28]. Thus, we report on youth-identified key aspects of the neighborhood´s built and social environments which include housing conditions, land vacancy, and community violence, and examine whether they are disproportionately concentrated in the neighborhoods with a greater share of Pittsburgh’s black population.

This multi-lens, mixed-method analysis was designed to challenge some of the assumptions we make about addressing inequality by using youths’ own opinions on the issue to drive our inquiry. Because black youth are disproportionately exposed to the double disadvantage of being poor and living in a poor neighborhood, learning from their experiences will help capture aspects of the neighborhood environment that may not be accounted for in large sample quantitative studies. Further, residents’ connections to place are under-examined in urban EJ scholarship and may play an important role in helping us understand how young people interpret and respond to environmental disparities [29].

### Background

Research suggests that poverty exerts a harmful effect not only through resource deprivation at the individual and family level, but also through a unique geography of poverty, which includes multiple other systems that people in poverty interact with, including neighborhoods [30,31]. The legacy of segregation in the United States has entrenched poor blacks in neighborhoods with reduced access to social capital and political services, and they are far more likely to live in (or near) neighborhoods characterized by hazardous environmental features [1,2,3,32,33,34,35,36] including factors such as pollution and toxic sites as well as dilapidated and dangerous housing, litter, disorder, and undesirable land uses [37]. Further, they are less likely to live in neighborhoods with desirable land uses and health-promoting amenities like parks, trees, and green spaces [38]. These visual environmental cues, in addition to their direct harmful effects (e.g., asthma, injury), have been associated with a variety of negative indirect outcomes, including anxiety, hopelessness, and depression [6,39]. EJ scholarship has shown that poor, racial and ethnic minority neighborhoods disproportionately bear the brunt of environmental harm in the United States [37,40] and it is well established that neighborhoods matter when it comes to explaining racial disparities in health and well-being [5,7,39,41]. Less clear, however, is what it is about neighborhoods that accounts for these disparities and, more importantly, how neighborhood environmental features can be changed to improve youth health and well-being [14,31].

In order to better understand these physical, environmental settings, the present study aimed to understand, from the perspective of young people themselves, how they interpret neighborhood environmental disparities. It is particularly important to learn from the direct perspectives of youth because, in addition to being physiologically and behaviorally more vulnerable to contaminants than adults, the research suggests that they, “have a unique way of understanding the proximal neighborhood environment that often eludes objective structural descriptions of a neighborhood” [19,42]. Youth may differ significantly in their descriptions of their neighborhoods from their parents and accounting for these different perspectives allows us to recognize the unique autonomy and opinions of young people, thereby creating a pathway through which youth can be given the opportunity to advocate for their neighborhoods [43,44]. Adolescents are more autonomous than younger children, whose parents may take measures to protect and supervise them within the neighborhood environment, and they are more likely than younger children to actively commute to school and take unsupervised journeys on foot through their neighborhoods [45,46]. These unique experiences make adolescents strong reporters on their neighborhood environment.

In addition to being a part of a marginalized population due to their race, the youth in the sample for this study were also part of several other uniquely vulnerable populations due to their gender, socioeconomic status, and neighborhood of origin. EJ draws on a civil rights framework and recognizes the relationship between race, place, space and the distribution of resources and hazards. Increasingly, research using an EJ framework has expanded to go beyond examining race and class to incorporating intersecting identities and multiple dimensions of inequality, including, for example, age and gender [47]. While we know that distressed neighborhood environments are characterized by more than poverty alone, and that the perceptions of neighborhood residents may matter even more for their outcomes than objectively measured neighborhood features, most studies rely on census-based measures of poverty and disadvantage (e.g., unemployment, education, and single-mother families) to characterize neighborhood level environmental inequality [48]. Few EJ studies specifically focus on the perspectives of youth (noteworthy exceptions include [17,18]), even though youth in these neighborhoods are perhaps the most likely to come across these incivilities in their daily routine and may have a great deal to contribute to our knowledge of the everyday experience of living in a poor, environmentally marginalized community [18,49]. It is important to go beyond income-based measures of poverty to understand what it is about a poor neighborhood that curtails life chances and leads to so many deleterious outcomes, and to identify what aspects might be malleable and thus addressed using locally relevant policy strategies.

## 2. Materials and Methods 

### 2.1. Study Setting

The research for this study takes place in Pittsburgh, Pennsylvania. Despite being named America’s most livable city by Forbes Magazine in 2014, Pittsburgh is home to stark racial disparities. Pittsburgh is primarily a black and white city, where less than 10% of youth residents between the ages of 5 and 17 years old are of other races. Davis and Bangs [50], who authored a study of racial demographics in Pittsburgh noted, “African Americans in our region remain at the bottom of every measure of the quality of life, which include indicators of economic status, educational achievement, family stability, and violence” (para. 1).

Using data from the American Community Survey (2005-09), we estimated the disparity in exposure to neighborhood poverty among Pittsburgh’s black and white urban youth. Black youth, both poor and non-poor, are more likely than white youth to live in high-poverty neighborhoods (i.e., neighborhoods where more than 30% of residents are below the poverty line). Even more startling is that a greater share of non-poor black youth (42%) live in these high-poverty neighborhoods than poor white youth (25%). Furthermore, only 3% of poor white children live in Pittsburgh’s highest (>40%) poverty neighborhoods, compared to nearly 40% of poor black children. These statistics suggest that Pittsburgh is an ideal place to study the racial inequality in neighborhood contexts among youth. Our qualitative data are drawn from a sample of youth who resided in Homewood, a low-income black community on the east end of Pittsburgh. The census tracts that make up Homewood are classified by the Pennsylvania Department of Environmental Protection as environmental justice areas. According to PA statutes, an EJ area is any census tract with more than 20% of individuals in poverty and/or 30% minority population [51]. Despite many institutional assets, Homewood is plagued by gun violence, high rates of school failure, and disproportionate social service systems involvement. Homewood’s built environment is among the most blighted in Pittsburgh [52]. More than half of the parcels (57%), including land and structures, are vacant and almost a quarter (22.3%) of all buildings in Homewood had code violations in 2010 [53]. Homewood residents experience multiple forms of marginalization along personal and environmental dimensions and, therefore, are a strong source of reporting on the experience of environmental disparities in this community.

### 2.2. Data Sources and Analysis

#### 2.2.1. Qualitative Data and Methods

We began by analyzing qualitative data collected in partnership with a group of black youth (age 14–19) from Homewood. This dataset was collected from 2010–2014 using a mixed-methods community-based participatory approach that included participatory photo mapping (*n* = 10), which combines photography and youth-led neighborhood tours; in-depth interviews with youth (*n* = 21), and spatial analysis [54]. The products of this research include more than 15 h of transcribed interviews, over 100 youth-authored photos of neighborhood strengths and weaknesses, youth-generated community maps, and presentations the youth created to highlight their research findings (The data reported in this study include qualitative comments made by the youth during in-depth interviews and other structured research activities. More detail on the participatory nature of the qualitative data collection and how photographic data were used can be found in previous publications [52,53]). Because of the unique, long-term engagement methods the first author utilized within the Homewood community, the qualitative data from this study were drawn solely from Homewood. The youth who made up this qualitative sample were members of two summer youth programs located in the Homewood community. The majority of the youth (*n* = 27) were in a summer program that included daily activities related to environmental issues such as litter clean ups and instruction on environmental sustainability. These youth were purposively sampled based on their participation in Homewood-based programs, and may have been more in tune with environmental issues than their peers due to their exposure to environmental education. The remaining youth (*n* = 4) were recruited from a program focused on academic enrichment.

These data were used to identify what aspects of low-income communities matter to youth and allowed us to translate youth-defined signs of poverty and environmental inequality into quantitative measures using neighborhood-level indicators data. 

We analyzed the qualitative data using a multi-cycle coding process. Coding is designed to reduce large amounts of data into small, meaningful labels to make connections between various concepts. We began the process with first cycle, or open coding, and created a codebook using ATLAS.ti 7.0 software (ATLAS.ti Scientific Software Development GmbH, Berlin, Germany) [55]. Using the existing codebook, we engaged in second cycle or axial coding, which was more focused and allowed us to reduce the data to represent the most essential trends [56,57]. The qualitative data allowed us to identify what aspects of poor, racially segregated communities had the largest detrimental impact on young people, and shaped our analysis of the quantitative data.

#### 2.2.2. Quantitative Data and Methods

We used the youth-identified themes to map the occurrence and concentration of indicators of neighborhood environmental inequality across Pittsburgh’s 90 neighborhoods (*n* = 141 census tracts). Data sources included demographic and poverty-related data from the American Community Survey (ACS) (2005-09). The administrative data were drawn from the Southwest Pennsylvania Community Profiles, which include a wide range of property-related neighborhood indicators at the land parcel level, including tax delinquency status, property ownership, and building inspection code violations obtained in their raw format through a data-sharing agreement [52]. It should be noted that due to changes in census geography between 2000 and 2010, some census tract boundaries within Pittsburgh no longer conformed to established neighborhood boundaries. Since some of the local data we utilize in this analysis come from before 2010, we opted to utilize the most recent data available prior to the change in census tract boundaries, which is the 2005–2009 ACS data. These data remained relatively constant across the city of Pittsburgh and therefore should adequately capture youths’ exposure to poverty and demographic variables during the time of data collection. While our data are Pittsburgh specific, similar measures are available in many cities across the U.S. through efforts like the Urban Institute’s National Neighborhood Indicators Partnership, so the study will be replicable and transferrable to other contexts. Locally collected neighborhood indicator data are particularly useful because they are collected at a more granular level than census data and can be used to better understand features of neighborhood context related to poverty and environmental disparities [58,59,60,61].

We used these data to map the intersection of income-based measures of poverty and youth-identified aspects of environmental poverty to descriptively show the distribution of these indicators. We began by creating descriptive bivariate choropleth maps, or maps that use different colors or shades to represent categories or classed values, to illustrate neighborhood demographic makeup, housing conditions, and occupancy.

Bivariate choropleth mapping is a technique in which you can visualize variation in two separate variables simultaneously [62]. Using ArcMap 10.3 (esri, Redlands, CA, USA), we created variables and obtained shapefiles for each of the youth-defined environmental indicators including race, poverty, housing vacancy, illegal dumpsites, and violent crime. We then created maps using a two-variable, three-class by three-class design to visualize the relationship between sets of two variables. We chose to visualize the data with a three-by-three class design to simplify visualization for the reader; cartographic conventions suggest that utilizing more than nine classes would risk making the classes more difficult to distinguish from one another [62,63]. We determined class data ranges for each variable using the “classify” tool in ArcMap 10.3, and then created new variables that separated each existing variable into three classes based on these data ranges. The classes were created using the “quantile” option in ArcMap 10.3 which separates the variable into classes based on three equal count breakpoints. The “field calculator” tool was then used to create a third variable that symbolizes the combination of the two variables (for example, poverty and race) by its position in the nine-class color scheme [62]. This new variable was used to populate each of the class combinations and allowed us to match each with its appropriate position and color in the sequential color scheme. We then used the “symbology” tool to visualize a nine-class sequential color scheme that included one color for each class combination [62,64].

We also used kernel density mapping techniques, which use color gradations to illustrate the spatial variations in the density of an attribute, to analyze hotspots of illegal dumpsites and whether they are spatially concentrated in low-income minority neighborhoods.

### 2.3. Ethical Statement

All subjects gave their informed consent for inclusion before they participated in the study. The study was conducted in accordance with the Declaration of Helsinki, and the protocol was approved by the Ethics Committee of the University of Pittsburgh (PRO11050246).

## 3. Results

Through the qualitative analysis, we uncovered several key features that defined the neighborhood environment in Homewood for young people. These youth-identified themes will be further described and situated within city-level administrative data to illustrate their prevalence and distribution across Pittsburgh neighborhoods. We begin by describing the relationship between race and poverty in Pittsburgh using a bivariate choropleth map. This description helps to frame the discussion of neighborhood environmental disparities as one of particular importance to poor black youth in the city of Pittsburgh. We then describe the results which highlight the most important themes defined by our youth participants including stereotypes about race and poverty and their relationship to neighborhood environmental disparities, high prevalence of vacant and deteriorated land and housing, symbols of disorder including litter, and exposure to violence. The results presented are descriptive in nature.

### 3.1. Race and Poverty

**Figure 1:**
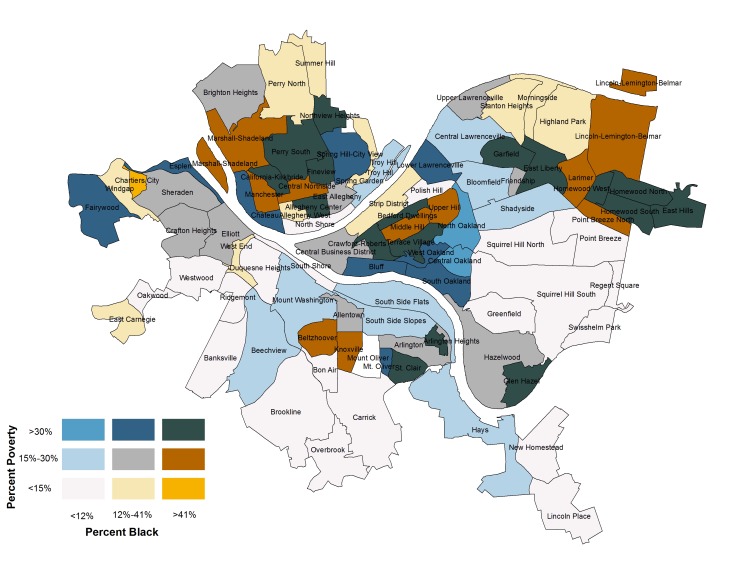
Map illustrating poverty and percent black across Pittsburgh neighborhoods.

In order to better understand the intersection of poverty and race in Pittsburgh, we created a bivariate choropleth map (see Figure 1). This map overlays the percent poverty with the percent black for each of Pittsburgh’s 90 neighborhoods, with the lightest colors reflecting low levels of poverty and a small black population (light grey, bottom left corner of legend), and the darkest colors representing the highest concentrations of both poverty and black population (dark blue, top right corner of legend).

As is the case in many communities, race and poverty are correlated in Pittsburgh. The correlation between percent black and percent poverty was 0.48 (*p* = 0.000). While there are 20 low-poverty, predominantly white neighborhoods (visualized in light grey), there is only one low-poverty predominantly black neighborhood in Pittsburgh (visualized in orange). Only two predominantly white neighborhoods in Pittsburgh are high poverty and these two neighborhoods are home to the University of Pittsburgh, where there is a large, transient college student population. In stark contrast, there are 17 high-poverty, predominantly black neighborhoods (visualized in dark blue).

The youth noted that Homewood and other predominantly black neighborhoods in Pittsburgh are stereotyped as being poor and disorderly. They used terms with distinct racial undertones such as “ghetto” and “ratchet” to describe how Homewood is viewed by the eyes of outsiders. For example, one youth noted, “A lot of people say it’s ratchet. Like ghetto. Loud. Crazy. Just always wantin’ to fight, always being rowdy and everything”. The areas of the neighborhood that the youth defined as good and safe were also defined in racial terms, but in terms of their perceived similarity to white neighborhoods. For example, one youth described his block, which is physically located within Homewood, “to me, it’s not that bad to live in Homewood. Cause, like, the place I live, like there’s nobody around it. It’s just like a white neighborhood”. The youth used racial descriptors to define different areas of the neighborhood and discussed how racial stereotypes shape outsiders’ perceptions of those who reside in Homewood:

The majority of Homewood is black people and people already think the mentality of black people are bad. Cause that’s all you hear about, gang bangin’ and that’s it. Gang bangin’ and shooting and drinkin’ and doin’ drugs. And that’s all they think that we’re about.

These quotes help provide context to better understand the experience of living in a neighborhood that is predominantly poor and black; a neighborhood that is highly stigmatized. Viewed from a social justice perspective, environmental justice issues are seen as part of the larger problems of racial, social, and economic justice and can be used to further describe the impact of race, politics, and class on quality of life [65]. The youth described the stigma and attitudes towards poor black individuals in general, and Homewood in particular, as one of the reasons they see disparities in the environmental factors described below.

### 3.2. Disorder

Another issue that the youth described is the perception that the city does not adequately care for public property and that residents of Homewood do not care about the physical condition of the neighborhood. The social context of neighborhoods dictates the amenities and services the neighborhood receives and is a crucial factor in the distribution of resources and risks in the city. Historically, black neighborhoods have been denied public amenities and today, segregated lower-income, minority neighborhoods may still receive lower-quality, less frequent municipal services [32,66]. Youth were attuned to environmental inequities and expressed frustration about the lack of response to litter and dumping by the city and adults in their community. They reported that the presence of litter and garbage further stigmatized the community and described how it affected the overall perception of Homewood and its residents. For example, one youth stated, “They basically like refer it on black people. Like, those black people are dirty, Homewood people are dirty, like they don’t take care of their neighborhood”. The youth noted the intersection between race and environmental issues, touching on the notion that when outsiders see litter and disorder, they see it as an embodiment of negative stereotypes about black neighborhoods [67]. Another, who had been involved in community clean-up activities, expressed frustration at her fellow community members for the neighborhood’s environmental conditions:

Like, I hate it how when we clean, like, it just gets dirty again. There will be a trash can on the corner and we’ll walk past and we’ll see a whole bunch of trash next to the garbage can and I’m like, are you serious? The trash can is right there.

**Figure 2:**
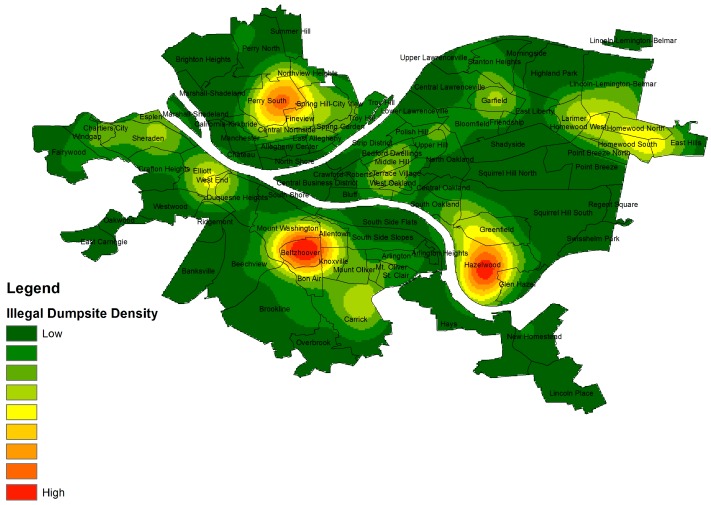
Heat map of illegal dumpsites across the city of Pittsburgh.

They described litter and waste as a major problem in the community and a visual indicator of both environmental problems and a lack of response to these problems. As a proxy for litter, we mapped the prevalence of illegal dumpsites in Pittsburgh neighborhoods. While illegal dumpsites are an imperfect proxy for the presence of trash and litter, and it is important to make the distinction between the etiology of litter [67] and illegal dumping [68], in our observations over the course of several years working in Homewood, Homewood was the site of both high levels of litter and illegal dumpsites. These data were obtained from Allegheny Cleanways, a Pittsburgh-based non-profit that works to eliminate and clean up illegal dumping and litter in Allegheny County, Pennsylvania. In their most recent report, Allegheny Cleanways described the most common types of waste in Pittsburgh’s illegal dumpsites as tires and household waste (particularly hard-to-dispose-of items such as televisions) [69]. The following map (Figure 2) uses kernel density mapping to create a “heat map” that shows the density of occurrence of illegal dumpsites across Pittsburgh. 

Though there was not a statistically significant correlation between the number of illegal dumpsites in a community and the percent of black residents, the map shows several hotspots with high density of illegal dumpsites. The neighborhoods surrounding Homewood show medium levels of illegal dumpsite density. The dumpsites, like the litter and garbage that the youth noted, confirm that the “hotspot” communities are largely unregulated by formal and informal control mechanisms. The youth reported that the city lacks a formal response to address dumping in their community and their fellow residents are not exercising informal control mechanisms or setting social norms that prevent litter and household waste dumping, leaving environmental hazards unchecked within the community. Housing abandonment is another feature noted by the youth that points to institutional and intra-neighborhood disinvestment and environmental degradation.

### 3.3. Housing Abandonment

Our qualitative data indicated that Homewood youth were concerned about the condition of the built environment in Homewood, particularly housing. They reported that much of the housing in Homewood is in a dilapidated condition and identified housing vacancy and abandonment as one of the most serious issues in Homewood [70]. They described vacant buildings and vacant land as not only an eyesore, but as a place in the community that facilitates crime, delinquency, and negative health behaviors:

(If) we were able to get like, crackheads and stuff, and get rid of these abandoned houses that they go in, like they’ll break into the abandoned houses. I could say, like if we didn’t have all these abandoned houses and people started moving here…Homewood would be a much better place.

The youth above described how abandoned houses attract drug users who go on to further damage the community. They also described how housing abandonment makes them feel. Many described abandoned housing as making them feel fearful, anxious, and sad. One youth stated, “Like, it has a great effect on your mood, you know? It just doesn’t make anything better. It’s just these open spaces filled with nothin”. They described the vacant lots left in the wake of razing abandoned homes as another environmental feature that cues negative emotions, promotes deleterious health behaviors, and stigmatizes their neighborhood. The presence of abandoned homes and lots stirred visceral responses in the youth, akin to the phenomenon of “root shock”, which is the traumatic stress associated with witnessing destruction and displacement [71].

**Figure 3:**
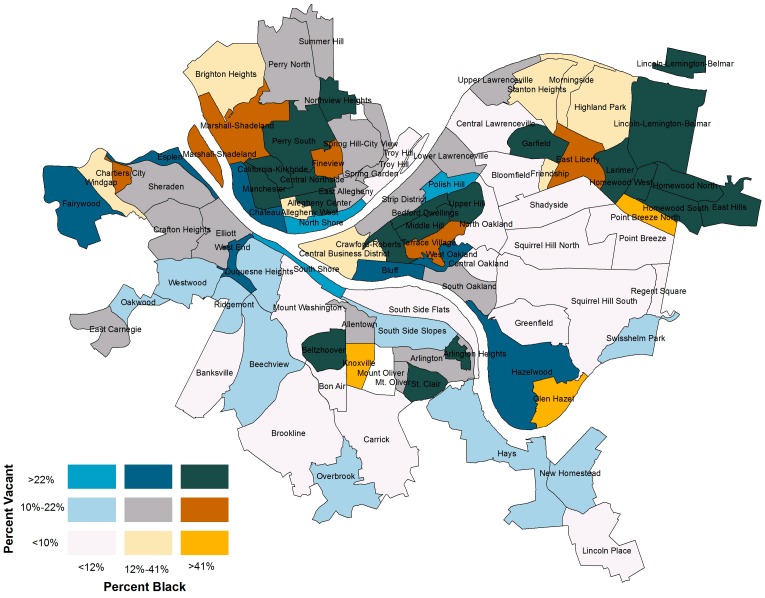
Map of vacancy by percent black across Pittsburgh neighborhoods.

Our maps indicate that housing vacancy is associated with neighborhood racial makeup. The following bivariate choropleth map (see Figure 3) illustrates the percent of total neighborhood parcels that are vacant alongside the percent of the neighborhood population that is black.

The youth noted that they felt that poor black communities like Homewood are marginalized by local government agencies and that people are forced to abandon their homes in the face of poverty and violence. The correlation between percent black and percent vacant was 0.61 (*p* = 0.00). While there are 19 predominantly white, low-vacancy neighborhoods (visualized in light grey), there are only three low-vacancy, predominantly black neighborhoods in Pittsburgh (visualized in orange). Only three predominantly white neighborhoods in Pittsburgh are high vacancy. In stark contrast, there are 21 high-vacancy, predominantly black neighborhoods (visualized in dark blue). The youth were also particularly attuned to the relationship between environmental features like housing abandonment and the prevalence of violent crime in Homewood.

### 3.4. Violent Crime

Environmental hazards in Homewood including overgrown vacant lots, abandoned houses that are open to entry, and a lack of formal and informal social control facilitate and compound issues of violent crime in Homewood. The youth in our Homewood-based sample reported extensively on how crime and violence mark their daily lives. The youth described fear of shootings and many had experienced or witnessed gun violence themselves. One young man described:

I seen my first shootout when I was in elementary…I was like, 10–15 feet away from it and I was watchin’ like I didn’t know what to do and I was just like watchin’ and my mom yelled at me, like, come in the house! (laughs)

In addition to describing lifelong exposure to crime and violence, they described how it changes the way they interact with their environment. Many, like the youth below, described the unpredictability of gun violence in Homewood:

If I’m riding (my bike) down the street I just don’t want to get shot on my bike for no reason “cause they’re trying to shoot somebody else…it is something I really do think about because…when me and my friend be ridin” out in the street we just be thinkin’ like, you never know what can happen.

**Figure 4:**
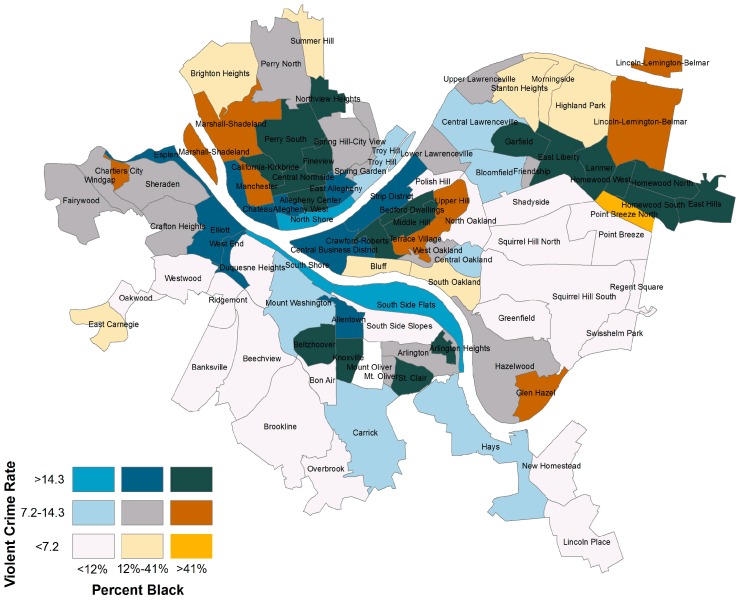
Map of violent crime rate by percent black across Pittsburgh neighborhoods.

This young man described how he and his friends fear riding their bikes because of the potential to be victimized. This has a variety of health implications, including the potential for serious injury if they are victimized as well as potential mental health issues related to fear and anxiety and the inability to safely exercise outdoors. They expressed worry for their safety and described feelings of helplessness related to unpredictable gun violence. In order to better illustrate the prevalence and concentration of violence in Pittsburgh, this bivariate choropleth map illustrates the relationship between the percent black population in Pittsburgh neighborhoods and the violent crime rate (see Figure 4).

The violent crime rate includes all Part 1 crimes against persons (homicide, sexual assault, robbery, aggravated assault) from 2005–2011. Rates were averaged between the years of 2005 and 2011 and were calculated by the number of instances per 1000 people based on population data from the 2010 census [52]. The map indicates that there are 21 low-violence, predominantly white neighborhoods whereas there is only one low-violence, predominantly black neighborhood. Conversely, 20 of the neighborhoods with the highest rates of violent crime are predominantly black and only three predominantly white neighborhoods have high levels of violent crime. Violent crimes appear to be concentrated in black neighborhoods in Pittsburgh. The youth reported a vicious cycle in which crime and violence drive residents to abandon their homes and leave Homewood. This then leaves the abandoned houses and lots to become sites that facilitate the commission of such crimes. Thus, violent crime compounds the issues of neighborhood stigma and drives environmental inequality and further exposure to neighborhood hazards.

## 4. Discussion

The present study examines racial inequality in the built and social neighborhood environment of urban youth, extending the prior literature in two key ways. First, we use youth perceptions to inform our understanding of what aspects of a neighborhood’s built and social environment shape youth well-being. Second, we use local level data to create indicators of the features of the neighborhood environment that youth describe as important (e.g., illegal dumping, vacancy, and violence). We map these features across Pittsburgh neighborhoods to examine racial disparities in exposure to these features of the neighborhood environment and to identify environmental inequities. The maps provide descriptive evidence to support the youths’ assertions that the environments of black and white individuals in Pittsburgh differ in noteworthy ways. The evidence suggests that environmental inequities are key visual indicators of inequality for youth.

The youth in this study identified environmental hazards that they believed were unique to Homewood and our maps illustrate that these issues were prevalent across black communities in Pittsburgh. They identified micro-level hazards [27] present in their neighborhoods and on their blocks, and described how it affected their perceptions of their neighborhood and outsiders’ perceptions and responses to the neighborhood. The youth recognized that Homewood residents are disproportionately exposed to negative environmental features and the lack of a formal response to the neighborhoods’ concerns left them to unanimously express the concern that no one cares about their neighborhood.

Our study is limited by the fact that our qualitative data are drawn from a sample of youth from one Pittsburgh neighborhood, which may not be representative of all EJ communities within the city of Pittsburgh. The first author spent several years building rapport and working in partnership with youth in Homewood, so it was not within the scope of this manuscript to expand the qualitative sample beyond this community. Future research should consider how youth from other EJ communities in Pittsburgh perceive environmental inequities to gain further insight from youth in other types of neighborhoods. 

## 5. Conclusions 

Our results suggest that the intersection of race and poverty, neighborhood disorder, housing abandonment, and crime are particularly salient issues for the youth in our qualitative sample. Our multi-lens, mixed-method analysis was designed to challenge some of the assumptions we make about addressing inequality using youths’ own opinions on the issue to drive our inquiry. It provides evidence to better understand what aspects of neighborhoods are important and may be addressed to promote safe and supportive neighborhood environments for youth. Currently, urban issues like housing discrimination, segregation, and issues in the built environment are often segregated into disciplinary silos outside of the environmental justice literature, despite the fact that they are deeply interconnected issues [72]. Future research can address this gap by taking an interdisciplinary approach to environmental justice research and carefully considering the opinions of residents, particularly young people, who are affected most by these issues.

This work lends itself to policy and practice-related implications. Our research may provide evidence to better understand what aspects of neighborhoods are important for young people’s well-being, and which of those aspects may be addressed to promote neighborhoods that support young people’s well-being. Future research can connect this evidence to interventions that may help non-profit organizations, local governments, and community groups to leverage funding that promotes community-driven interventions that support residents’ needs and address features of neighborhood inequality like the built and natural environment. For example, this research can help target programs and policies that eliminate neighborhood blight, provide support to stabilize existing residents in their homes (e.g., through tax abatement, etc.), and provide a guide for improving the physical environment of communities without displacement. 

The environmental justice movement is beginning to tackle the challenges of displacement, gentrification, and built environmental issues in urban neighborhoods [26,73]. Some promising approaches include improving community engagement, particularly among youth. Though the contributions of children and youth to community movements have been noted in the literature, their opinions are still sought less frequently than those of adults. Engaging youth in addressing community-level environmental disparities can have the dual positive impact of helping youth developmentally/interpersonally and helping to improve the community [74]. Promoting environmental literacy among young people can also improve health literacy and, in effect, empower youth to address environmental health disparities [75]. Multiple approaches that attend to the intersections of social, economic, and environmental justice issues might be necessary. While one intervention may be necessary to target issues in the built and physical environment, others may simultaneously target social connections, the experience of neighborhood stigma, and being cut off from city services, for example [10]. 

One youth from our qualitative sample in Homewood stated, “I just think like, if you could be raised here and make it out, you can do anything. It’s just a big motivation for me, like I know where I don’t want to end up”. In many urban neighborhoods, one of the hallmarks of youth success is leaving the neighborhood.

Programs aimed at reducing environmental health hazards can help create communities where people can stay and thrive, through responsible redevelopment and policies that stabilize existing residents and reduce environmental features that facilitate crime and violence.
